# Concomitant Immunosuppressive Therapy Use in Eculizumab-Treated Adults With Generalized Myasthenia Gravis During the REGAIN Open-Label Extension Study

**DOI:** 10.3389/fneur.2020.556104

**Published:** 2020-11-24

**Authors:** Richard J. Nowak, Srikanth Muppidi, Said R. Beydoun, Fanny L. O'Brien, Marcus Yountz, James F. Howard

**Affiliations:** ^1^Department of Neurology, Yale University School of Medicine, New Haven, CT, United States; ^2^Department of Neurology and Neurosciences, Stanford University School of Medicine, Stanford, CA, United States; ^3^Department of Neurology, University of Southern California, Los Angeles, CA, United States; ^4^Alexion Pharmaceuticals, Boston, MA, United States; ^5^Department of Neurology, University of North Carolina, Chapel Hill, NC, United States

**Keywords:** eculizumab, myasthenia gravis, immunosuppressive therapy, refractory, acetylcholine receptor

## Abstract

**Introduction:** Chronic, broad-spectrum immunosuppressive therapy (IST) can be associated with side effects in many people with generalized myasthenia gravis (gMG), and treatment guidelines recommend that the IST dose be tapered once patients achieve a stable treatment response. We therefore examined IST use in eculizumab-treated patients with refractory gMG.

**Methods:** The REGAIN open-label extension (OLE) enrolled 117 adults with refractory anti-acetylcholine receptor antibody-positive gMG who had completed the 6-month, randomized, double-blind, placebo-controlled REGAIN study of eculizumab. Eligible patients had received ≥2 ISTs for ≥1 year or ≥1 IST with intravenous immunoglobulin or plasma exchange ≥4 times in 1 year, without symptom control. During REGAIN, changes in concomitant MG therapies were not permitted; during the OLE, they were permitted at the investigators' discretion. Participants received eculizumab 1,200 mg every 2 weeks for up to 4 years; concomitant prednisone and related corticosteroids (PRED), azathioprine (AZA), and mycophenolate mofetil (MMF) use was recorded. Changes in MG Activities of Daily Living and Quantitative MG total scores, MG exacerbations, and adverse events were also recorded.

**Results:** At last OLE assessment, 88.0% (103/117) of participants were using ≥1 IST vs. 98.3% (115/117) at OLE baseline. During the OLE, 76.9% (90/117) of patients experienced a total of 719 IST changes. Almost half of participants [48.7% (57/117)] stopped or decreased ≥1 IST owing to MG symptom improvement, representing 38.9% (280/719) of all changes. In patients who decreased and/or stopped ≥1 IST, mean daily doses of PRED, AZA, and MMF decreased between OLE baseline and last assessment by 60.8% [standard deviation (SD), 28.07; *P* < 0.0001], 89.1% (SD, 25.77; *P* < 0.0001), and 56.0% (SD, 32.99; *P* < 0.0001), respectively. Improved clinical outcomes were observed with eculizumab regardless of IST changes during the OLE, and eculizumab's safety profile was similar in patients who used PRED, AZA, and MMF.

**Conclusions:** Use of ISTs by patients with previously refractory gMG decreased during eculizumab treatment in the REGAIN OLE. Clinical improvements with eculizumab were maintained by patients in all groups, including those who decreased and/or stopped concomitant ISTs.

**Trial registration:** www.clinicaltrials.gov: NCT01997229, NCT02301624.

## Introduction

Generalized myasthenia gravis (gMG) is an immune-mediated neuromuscular disorder characterized by fatigable muscle weakness. Most patients with MG (70–88%) have autoantibodies to the acetylcholine receptor (AChR) (1–6). These autoantibodies cause accelerated degradation of AChRs and activation of the complement cascade, resulting in structural damage to the neuromuscular junction (7–13) and thus impairing neuromuscular transmission and muscle strength (7–9, 14).

The current guidelines for the management of MG recommend that immunosuppressive therapy (IST), including prednisone and related corticosteroids (PRED), should be used in all patients with MG who have not met treatment goals after an adequate trial of pyridostigmine (15). Most patients with MG receive long-term IST (16), but use of these treatments is often associated with unwanted effects (especially in the case of prolonged use) and may adversely impact quality of life (17, 18). It is recommended that the IST dose be tapered once patients experience a stable response (15), an approach that is favored by both clinicians and patients (19). Approximately 10–15% of patients with MG have refractory disease on the basis that they do not respond adequately to ISTs, they require maintenance intravenous immunoglobulin or plasma exchange treatment, or they experience intolerable adverse events associated with ISTs (15, 20, 21).

REGAIN, a 6-month, phase 3, randomized, placebo-controlled study, and its open-label extension (OLE) demonstrated rapid and sustained efficacy and tolerability of the terminal complement inhibitor eculizumab in adults with refractory anti-AChR antibody-positive (AChR+) gMG (14, 22). During REGAIN, participants continued their previously established IST regimens with no changes permitted (14). However, adjustment of concomitant MG therapies, such as ISTs (including PRED), was permitted at the discretion of the investigator during the OLE (22).

This analysis examined changes in the use of ISTs, including PRED, azathioprine (AZA), and mycophenolate mofetil (MMF), in patients receiving eculizumab during the OLE of the REGAIN study. Clinical outcomes in subgroups of patients defined by changes in IST use were also examined.

## Methods

### Study Design and Participants

REGAIN was a 6-month (26-week), phase 3, randomized, placebo-controlled clinical trial that assessed the efficacy and tolerability of eculizumab in patients aged 18 years or older with refractory AChR+ gMG (www.clinicaltrials.gov: NCT01997229) (14). In addition to confirmed AChR+ gMG and an MG Activities of Daily Living (MG-ADL) total score ≥6, eligible patients had to have refractory disease: they must have received two or more ISTs for at least 1 year or one or more ISTs with intravenous immunoglobulin or plasma exchange treatment at least four times in 1 year, without symptom control. Full eligibility and exclusion criteria have been published previously (14). Within 2 weeks of completing REGAIN, participants could enroll in the OLE (www.clinicaltrials.gov: NCT02301624) to receive open-label eculizumab for up to a maximum of 4 years. All participants were required to have received *Neisseria meningitidis* vaccinations at least 2 weeks before the first dose of study drug (or prophylactic antibiotics until 2 weeks after vaccination) and to be revaccinated according to local guidelines. The first patient was enrolled in the REGAIN study on April 30, 2014, and the extension study was completed in January 2019 (22). Data reported here are from the final follow-up of all patients in the OLE.

All patients provided written, informed consent. Independent ethics committees or institutional review boards provided written approval for the study protocols and all amendments.

### Treatment

Eculizumab and placebo administration during REGAIN and the OLE have been described previously (14, 22). During the OLE, participants received open-label eculizumab 1,200 mg every 2 weeks for up to 4 years after a 4-week blinded induction period.

During REGAIN, patients who had previously received ISTs were required to maintain their pre-study IST type, dose, and schedule (14). During the OLE, modifications to IST type, dose, and schedule were permitted at the investigators' discretion, although they were not required by the study protocol (22).

Concomitant ISTs included, but were not limited to, PRED (prednisone, prednisolone, methylprednisolone, methylprednisolone sodium succinate, and meprednisone), AZA, and MMF.

### Assessments

Use and dosages of concomitant ISTs, including PRED, AZA, and MMF, were reported at all scheduled visits, and at unscheduled visits for MG crises/exacerbations, from OLE baseline (day 1) to last assessment (study discontinuation or end of study). PRED doses and dose changes were expressed as prednisone equivalents: doses of methylprednisolone sodium succinate, methylprednisolone, and meprednisone were converted to prednisone equivalents by multiplying them by 1.25. The numbers of change events during the OLE, as well as the nature of and reasons for these changes, were reported for PRED, AZA, and MMF. Due to the small numbers of participants using other ISTs during REGAIN and the OLE (cyclosporine, tacrolimus, methotrexate, and cyclophosphamide; *n* ≤ 17 for each), change events for these ISTs during the OLE were not included in this analysis.

Changes in MG-ADL and Quantitative MG (QMG) mean total scores from eculizumab start (REGAIN baseline for eculizumab/eculizumab group and OLE baseline for placebo/eculizumab group) to last assessment were evaluated for all patients. The proportions of patients with exacerbations that did and did not meet the protocol definition [MG crisis, significant symptomatic worsening (an increase either by 2 points or to a score of 3 for any single MG-ADL item, excluding ocular items), or health in jeopardy without rescue therapy according to the treating physician] or who required rescue therapy were also recorded over this time frame.

Adverse events were recorded and coded by preferred term using the Medical Dictionary for Regulatory Activities Version 20.1.

### Statistical Analysis

Analyses were largely based on descriptive data. Mean percentage changes in IST doses were analyzed using one-sample *t*-tests, and median percentage changes in IST doses were analyzed using the Wilcoxon signed-rank test.

## Results

### Patient Disposition and Characteristics at Open-Label Extension Baseline

Nearly all (117/118) patients who completed REGAIN continued into the OLE (eculizumab/eculizumab, 56; placebo/eculizumab, 61) and were included in the efficacy and safety analyses (22). Of these, 87 patients completed the OLE (eculizumab/eculizumab, 43; placebo/eculizumab, 44), and 30 discontinued (eculizumab/eculizumab, 13; placebo/eculizumab, 17) owing to adverse events (*n* = 7), death (*n* = 3), patient withdrawal (*n* = 13), withdrawal by physician (*n* = 6), or “other” reason (*n* = 1) (23). The median duration of eculizumab treatment from OLE baseline to last assessment was 972.0 days (range, 1–1,372 days).

Patient demographics at OLE baseline have been reported previously (22). Baseline demographics were similar between groups of patients using PRED, AZA, or MMF at baseline, except that smaller proportions of Asian than white patients used AZA or MMF (Table 1). Also, patients receiving AZA at baseline showed a tendency to have previously used fewer ISTs than those receiving PRED or MMF, while prior plasma exchange was more common in those receiving MMF than in those receiving PRED or AZA (Table 1).

**Table 1:** Demographic and disease characteristics by concomitant immunosuppressive therapy (IST) in patients using prednisone and related corticosteroids (PRED), azathioprine (AZA), or mycophenolate mofetil (MMF) at open-label extension (OLE) baseline.

**Characteristic**	**PRED**	**AZA**	**MMF**	**All patients**
	***n* = 90**	***n* = 39**	***n* = 30**	***N* = 117**
**Age^a^, mean (SD), years**	48.3 (16.52)	46.7 (16.87)	49.4 (17.52)	47.4 (16.70)
**Sex**, ***n*** **(%)**				
Male	34 (37.8)	14 (35.9)	9 (30.0)	38 (32.5)
Female	56 (62.2)	25 (64.1)	21 (70.0)	79 (67.5)
**Race**, ***n*** **(%)**				
Asian	18 (20.0)	2 (5.1)	1 (3.3)	19 (16.2)
Black/African American	0 (0.0)	0 (0.0)	1 (3.3)	2 (1.7)
White	67 (74.4)	34 (87.2)	26 (86.7)	88 (75.2)
Unknown	1 (1.1)	1 (2.6)	0 (0.0)	1 (0.9)
Multiple	0 (0.0)	0 (0.0)	0 (0.0)	1 (0.9)
Other	4 (4.4)	2 (5.1)	2 (6.7)	6 (5.1)
**Region**, ***n*** **(%)**				
North America	31 (34.4)	16 (41.0)	11 (36.7)	43 (36.8)
South America	9 (10.0)	7 (17.9)	1 (3.3)	12 (10.3)
Europe	34 (37.8)	15 (38.5)	18 (60.0)	46 (39.3)
Asia Pacific	5 (5.6)	1 (2.6)	0 (0.0)	5 (4.3)
Japan	11 (12.2)	0 (0.0)	0 (0.0)	11 (9.4)
**Duration of MG^b^, mean (SD), years**	9.87 (8.13)	9.67 (8.17)	10.31 (8.59)	10.21 (8.23)
**MGFA classification by randomization stratification at screening**, ***n*** **(%)**				
IIa or IIIa	47 (52.2)	21 (53.8)	18 (60.0)	58 (49.6)
IVa	3 (3.3)	3 (7.7)	1 (3.3)	6 (5.1)
IIb or IIIb	36 (40.0)	13 (33.3)	10 (33.3)	47 (40.2)
IVb	4 (4.4)	2 (5.1)	1 (3.3)	6 (5.1)
**Prior IST use**, ***n*** **(%)**				
2 ISTs only	42 (46.7)	32 (82.1)	9 (30.0)	52 (44.4)
3 ISTs only	27 (30.0)	5 (12.8)	14 (46.7)	39 (33.3)
≥4 ISTs	20 (22.2)	2 (5.1)	6 (20.0)	24 (20.5)
**Prior IVIg use**, ***n*** **(%)**	70 (77.8)	29 (74.4)	24 (80.0)	92 (78.6)
**Prior plasma exchange use**, ***n*** **(%)**	39 (43.3)	17 (43.6)	17 (56.7)	57 (48.7)

*A total of 90 patients were using PRED at OLE baseline, 39 were using AZA, and 30 were using MMF. PRED, AZA, and MMF could be used as monotherapies, in combination with each other, or with other ISTs*.

a *On day 1 of OLE*.

b *Time from MG diagnosis to first dose date in the OLE*.

*IVIg, intravenous immunoglobulin; MG, myasthenia gravis; MGFA, Myasthenia Gravis Foundation of America; SD, standard deviation*.

### Changes in Immunosuppressive Therapy Use During the Open-Label Extension

At OLE baseline, 98.3% (115/117) of patients were using at least one IST. During the OLE, 99.1% (116/117) of patients used at least one IST at some point. At the last assessment, 88.0% (103/117) of patients were using at least one IST.

Over three quarters [76.9% (90/117)] of patients experienced a total of 719 change events in their IST regimens during the OLE. Stopping an IST or decreasing the dose of at least one IST [68.0% (489/719) of changes] was more common than starting an IST or increasing the dose of at least one IST [32.0% (230/719) of changes; Table 2]. In total, 71.8% (84/117) of participants stopped or decreased the daily dose of at least one IST at some point during the OLE. The most common reason for stopping or decreasing the dose was MG symptom improvement [48.7% (57/117) of participants on 280/489 occasions]. Conversely, among the 71/117 (60.7%) participants who started an IST or increased the daily dose of at least one IST at some point during the OLE, the most common reason for the change was MG symptom worsening [37.6% (44/117) of participants on 111/230 occasions; Table 2].

**Table 2:** Changes in immunosuppressive therapy (IST) use during the open-label extension.

**Type of/reason for change in IST use**	**IST change events, *n* (%)^a^*N* = 719**	**Patients, *n* (%)^b^*N* = 117**
Change in IST use	719 (100.0)	90 (76.9)
Decrease in daily dose of 1 IST	386 (53.7)	74 (63.2)
Increase in daily dose of 1 IST	158 (22.0)	51 (43.6)
Stoppage of an existing IST	101 (14.0)	51 (43.6)
Start a new IST	69 (9.6)	37 (31.6)
Increase in daily dose of >1 IST	3 (0.4)	3 (2.6)
Decrease in daily dose of >1 IST	2 (0.3)	2 (1.7)
Stoppage or decrease in dose of ≥1 IST	489 (68.0)	84 (71.8)
MG symptoms improved	280 (38.9)	57 (48.7)
MG symptoms worsened	3 (0.4)	3 (2.6)
New indication other than MG for IST use	13 (1.8)	7 (6.0)
Side effects—intolerant to existing IST	49 (6.8)	18 (15.4)
Other^c^	144 (20.0)	47 (40.2)
Start or increase in dose of ≥1 IST^d^	230 (32.0)	71 (60.7)
MG symptoms worsened	111 (15.4)	44 (37.6)
New indication other than MG for IST use	19 (2.6)	12 (10.3)
Other^c^	98 (13.6)	43 (36.8)

a *Percentage of all changes in IST use*.

b *Any given patient may have experienced multiple changes under the same or different categories*.

c *“Other” category largely used to describe temporary dosing/treatment changes in response to conditions such as asthma/chronic obstructive pulmonary disease, conjunctivitis, and urinary tract infections, or to support surgery*.

d *Two reasons in this category were problematic (not applicable values)*.

*MG, myasthenia gravis*.

### Changes in Prednisone and Related Corticosteroids, Azathioprine, and Mycophenolate Mofetil Use Between Open-Label Extension Baseline and Last Assessment

**Figure 1:**
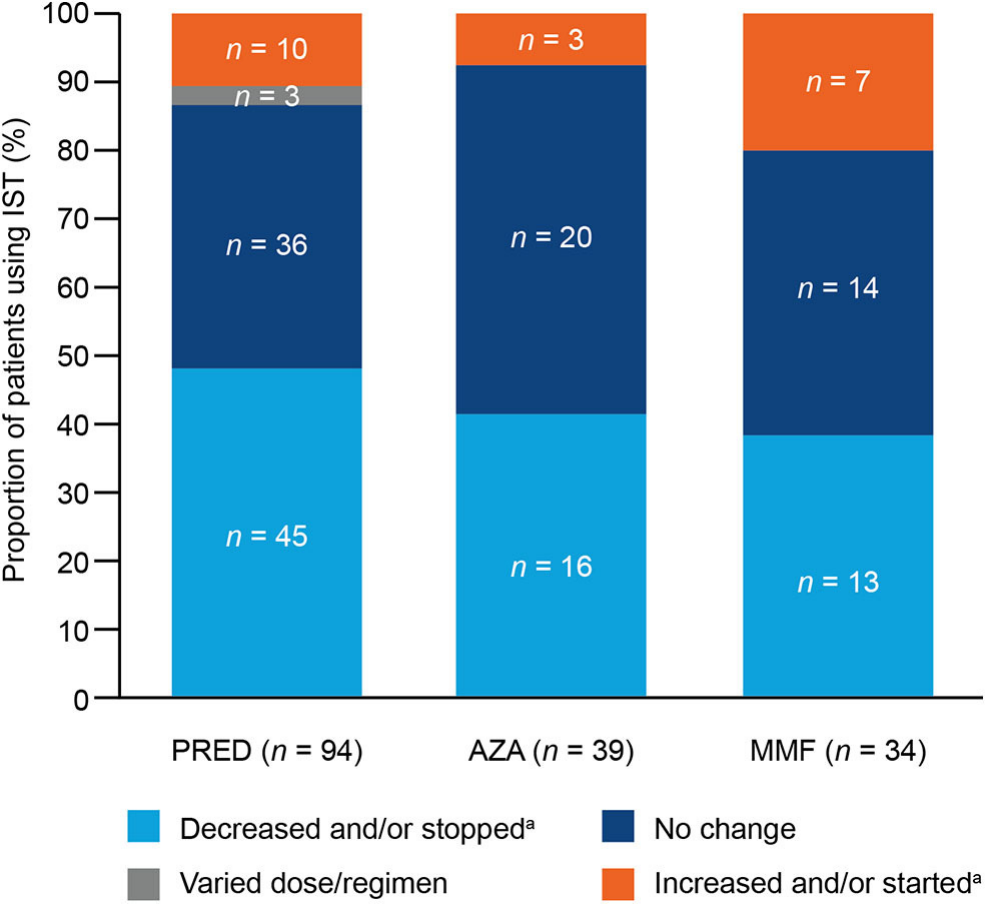
Overall changes to immunosuppressive therapy (IST) at any time during the open-label extension (OLE) in patients using prednisone and related corticosteroids (PRED), azathioprine (AZA), or mycophenolate mofetil (MMF). During the OLE, 94 patients used PRED (90 patients at OLE baseline), 39 patients used AZA (39 patients at OLE baseline), and 34 patients used MMF (30 patients at OLE baseline). ^a^Increases/decreases were calculated from the starting dose and the dose at the last assessment.

At OLE baseline, PRED were the most commonly used ISTs [being used by 90/117 (76.9%) patients], followed by AZA [39/117 (33.3%) patients], and then MMF [30/117 (25.6%) patients]. PRED and AZA were used in combination by 30/117 (25.6%) patients, and PRED and MMF were used in combination by 22/117 (18.8%) patients. Compared with those at OLE baseline, there were fewer patients at the last assessment using PRED [76/117 (65.0%)], AZA [26/117 (22.2%)], PRED and AZA combined [16/117 (13.7%)], and PRED and MMF combined [18/117 (15.4%)], with little change in the number using MMF [31/117 (26.5%)]. Most patients who used PRED, AZA, or MMF during the study had decreased and/or stopped or had no changes in their doses at the last assessment (Figure 1).

**Figure 2:**
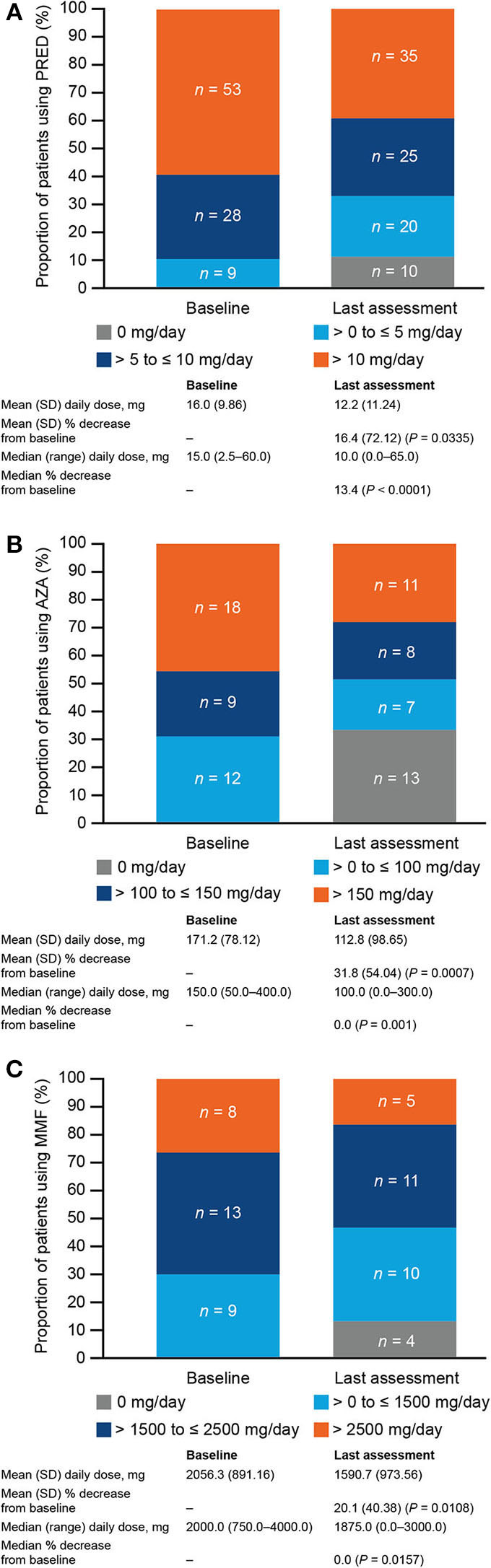
Immunosuppressive therapy (IST) doses at open-label extension (OLE) baseline and last assessment in patients using **(A)** prednisone and related corticosteroids (PRED; *n* = 90), **(B)** azathioprine (AZA; *n* = 39), or **(C)** mycophenolate mofetil (MMF; *n* = 30) at OLE baseline. A total of 90 patients were using PRED at OLE baseline, 39 were using AZA, and 30 were using MMF. The distribution of IST doses at OLE baseline and last assessment, mean and median daily doses at OLE baseline and last assessment, and mean and median dose reductions from OLE baseline to last assessment are shown for these patients. *P*-values for mean percentage changes were calculated using the one-sample *t*-test; *P*-values for median percentage changes were calculated using the Wilcoxon signed-rank test. SD, standard deviation.

Of the patients who used PRED during the OLE, almost half [47.9% (45/94)] decreased and/or stopped their PRED dose, and 38.3% (36/94) had no change in dose (Figure 1). At the last assessment, 10 of the 90 (11.1%) patients who had been using PRED at baseline were no longer using PRED (Figure 2A). Of the patients using PRED at baseline, the proportion using more than 10 mg of PRED per day decreased from 58.9% (53/90) at baseline to 38.9% (35/90) at the last assessment. Significant reductions in mean daily PRED dose from OLE baseline to last assessment were observed among all patients [16.4% (SD, 72.12; *P* = 0.0335); 3.8 mg/day (SD, 10.89; *P* = 0.0014); Figure 2A] and among those who decreased and/or stopped PRED [60.8% (SD, 28.07; *P* < 0.0001); 10.5 mg/day (SD, 7.49; *P* < 0.0001)].

Of the patients who used AZA during the OLE, 41.0% (16/39) decreased and/or stopped their AZA dose, and 51.3% (20/39) had no change in dose (Figure 1). One third [13/39 (33.3%)] of patients who had been using AZA at baseline were no longer using AZA at the last assessment (Figure 2B). Of the patients using AZA at baseline, the proportion using more than 150 mg of AZA per day decreased from 46.2% (18/39) at baseline to 28.2% (11/39) at the last assessment. Significant reductions in mean daily AZA dose from OLE baseline to last assessment were observed among all patients [31.8% (SD, 54.04; *P* = 0.0007); 58.3 mg/day (SD, 96.88; *P* = 0.0006); Figure 2B] and among those who decreased and/or stopped AZA [89.1% (SD, 25.77; *P* < 0.0001); 151.6 mg/day (SD, 87.31; *P* < 0.0001)].

Of the patients who used MMF during the OLE, 38.2% (13/34) decreased and/or stopped their MMF dose, and 41.2% (14/34) had no change in dose (Figure 1). Four of the 30 (13.3%) patients who had been using MMF at OLE baseline were no longer using MMF at the last assessment (Figure 2C). Of the patients using MMF at baseline, the proportion using more than 2,500 mg of MMF per day decreased from 26.7% (8/30) at baseline to 16.7% (5/30) at the last assessment. Significant reductions in mean daily MMF dose from OLE baseline to last assessment were observed among all patients [20.1% (SD, 40.38; *P* = 0.0108); 465.7 mg/day (SD, 872.48; *P* = 0.0067); Figure 2C] and among those who decreased and/or stopped MMF [56.0% (SD, 32.99; *P* < 0.0001); 1,228.5 mg/day (SD, 788.58; *P* < 0.0001)].

### Clinical Outcomes in Patients With Changes in Immunosuppressive Therapy Between Eculizumab Start and Open-Label Extension Last Assessment

Mean MG-ADL and QMG total scores decreased between the start of eculizumab therapy (REGAIN baseline for eculizumab/eculizumab group and OLE baseline for placebo/eculizumab group) and OLE last assessment across most groups of patients defined by change in IST use during the OLE (Table 3). The only exceptions to this were for changes in mean QMG total score in patients who increased and/or started PRED (0.2 increase; *n* = 10) and for changes in mean MG-ADL total score in those who increased and/or started AZA (0.3 increase; *n* = 3).

**Table 3:** Mean changes in Myasthenia Gravis Activities of Daily Living (MG-ADL) and Quantitative MG (QMG) total scores from eculizumab start^a^ to open-label extension (OLE) last assessment by immunosuppressive therapy (IST) and nature of dose change during the OLE.

**IST**	**Nature of dose change during the OLE**	**MG-ADL total score**		**QMG total score**	
		**Eculizumab baseline^a^, mean (SD)**	**Change to OLE last assessment, mean (SD)**	**Eculizumab baseline^a^, mean (SD)**	**Change to OLE last assessment, mean (SD)**
Total	All patients, *N* = 117	8.9 (3.60)	−3.6 (4.14)	15.9 (5.69)	−4.1 (5.81)
PRED^b^	Patients who decreased and/or stopped, *n* = 45	8.6 (3.57)	−4.7 (3.92)	16.0 (5.49)	−5.6 (5.15)
	Patients with no change, *n* = 36	8.9 (3.59)	−2.3 (4.11)	15.4 (5.49)	−1.5 (4.98)
	Patients who increased and/or started, *n* = 10	8.7 (3.16)	−0.7 (4.16)	14.9 (5.65)	0.2 (4.92)
AZA^b^	Patients who decreased and/or stopped, *n* = 16	7.6 (3.08)	−3.4 (4.00)	15.3 (4.61)	−3.8 (6.76)
	Patients with no change, *n* = 20	9.0 (4.15)	−4.7 (3.77)	16.0 (6.35)	−5.1 (5.26)
	Patients who increased and/or started, *n* = 3	7.7 (6.66)	0.3 (2.31)	13.3 (8.02)	−2.7 (4.93)
MMF^b^	Patients who decreased and/or stopped, *n* = 13	8.5 (2.57)	−5.1 (3.64)	14.1 (2.36)	−4.9 (3.52)
	Patients with no change, *n* = 14	9.0 (2.75)	−2.5 (3.37)	16.1 (4.92)	−1.6 (4.01)
	Patients who increased and/or started, *n* = 7	12.6 (2.23)	−5.3 (3.55)	20.0 (6.81)	−7.9 (5.24)

a *Eculizumab baseline is REGAIN baseline for the eculizumab/eculizumab group and OLE baseline for the placebo/eculizumab group*.

b *PRED, AZA, and MMF could be used as monotherapies, in combination with each other, or with other ISTs*.

*AZA, azathioprine; MMF, mycophenolate mofetil; PRED, prednisone and related corticosteroids; SD, standard deviation*.

During the OLE, 27 patients experienced protocol-defined exacerbations, and five patients experienced exacerbations that did not meet the protocol definition. Of these patients, nine experienced protocol-defined exacerbations after decreasing and/or stopping PRED, AZA, or MMF; and two experienced exacerbations that did not meet the protocol definition following a decrease in PRED dose. Additionally, one patient experienced a protocol-defined exacerbation following a decrease in cyclosporine dose.

### Safety

Eculizumab was well tolerated during both REGAIN and its OLE (14, 22). The most common adverse events that occurred in patients receiving eculizumab during these two studies were headache (44.4%) and nasopharyngitis (38.5%) (23). One meningococcal infection, which was resolved with antibiotic treatment, was reported during the OLE; three deaths occurred in patients with important comorbidities (22). The proportions of patients who experienced treatment-emergent adverse events were similar between groups of patients who used PRED, AZA, and MMF during the OLE (Table 4).

**Table 4:** Treatment-emergent adverse events by concomitant immunosuppressive therapy during the open-label extension (OLE) in patients using prednisone and related corticosteroids (PRED), azathioprine (AZA), or mycophenolate mofetil (MMF) at OLE baseline.

	**PRED**	**AZA**	**MMF**	**All patients**
	***n* = 90**	***n* = 39**	***n* = 30**	***N* = 117**
Total patients with events, *n* (%)	87 (96.7)	38 (97.4)	29 (96.7)	114 (97.4)
**Treatment-emergent adverse events occurring in >15% of all patients, *n* (%)**				
Headache	34 (37.8)	17 (43.6)	9 (30.0)	47 (40.2)
Nasopharyngitis	34 (37.8)	8 (20.5)	9 (30.0)	42 (35.9)
Diarrhea	17 (18.9)	14 (35.9)	7 (23.3)	29 (24.8)
MG^a^	23 (25.6)	10 (25.6)	12 (40.0)	29 (24.8)
Upper respiratory tract infection	21 (23.3)	15 (38.5)	6 (20.0)	28 (23.9)
Arthralgia	18 (20.0)	10 (25.6)	5 (16.7)	23 (19.7)
Cough	13 (14.4)	8 (20.5)	5 (16.7)	22 (18.8)
Influenza	15 (16.7)	9 (23.1)	6 (20.0)	22 (18.8)
Nausea	16 (17.8)	9 (23.1)	6 (20.0)	22 (18.8)
Urinary tract infection	9 (10.0)	4 (10.3)	6 (20.0)	19 (16.2)
Pain in extremity	14 (15.6)	8 (20.5)	3 (10.0)	18 (15.4)

*The number (%) of patients who experienced treatment-emergent adverse events is provided for each group. A total of 90 patients were using PRED at OLE baseline, 39 were using AZA, and 30 were using MMF*.

a *Worsening (increased frequency and/or intensity) of a pre-existing condition, including MG, is considered to be an adverse event*.

*MG, myasthenia gravis*.

## Discussion

### Immunosuppressive Therapy Use During the REGAIN Open-Label Extension

There is a burden associated with the use of ISTs in gMG (17, 18); it is therefore important to better understand what impact the addition of the complement inhibitor eculizumab may have on their use. In the REGAIN OLE, physician-directed changes in IST use were permitted. The present analysis demonstrates that concomitant IST use decreased during the OLE, over a median of 32 months. More patients stopped or decreased the dose of an IST than started or increased the dose of an IST; by the last assessment, over 10% of patients had stopped using concomitant ISTs. From baseline to the last assessment, the mean daily doses of PRED, AZA, and MMF were significantly reduced.

During eculizumab treatment in REGAIN and its OLE, improvements were observed in both patient-reported activities of daily living (MG-ADL total score) and physician-evaluated neurologic function related to MG (QMG total score) (14, 22). In this analysis, we sought to examine whether IST use impacts this response. We found that improvements were experienced regardless of the type of IST used or the nature of IST dosing change during the OLE. These improvements are notable considering that, at the start of REGAIN, all study participants had treatment-refractory gMG.

The long-term safety and tolerability of eculizumab have been reported from over 10 years of clinical use in atypical hemolytic uremic syndrome and paroxysmal nocturnal hemoglobinuria (24–28). Safety data from the final analysis of the OLE were consistent with interim OLE safety data and the known safety profile of eculizumab in gMG (14, 22). Incidences of adverse events during the OLE were similar between patients who used PRED, AZA, and MMF.

### Limitations of the Study

All adjustments of concomitant ISTs during the OLE were at the discretion of study investigators, with no protocol-specified procedures for IST tapering. IST changes during the OLE were therefore likely to have been heterogeneous, making it difficult to draw conclusions about whether clinical outcomes associated with them reflect patients' changing needs or the way in which the changes were implemented.

This analysis was largely based on descriptive data, and although the analysis of changes in IST use was pre-specified for the OLE, reasons for IST changes, clinical outcomes in patients with IST changes, and safety outcomes by IST type were analyzed *post-hoc*. The open-label design of the extension study is also a limitation of this analysis; however, selection or reporting biases are unlikely because over 90% of REGAIN participants continued into the OLE.

### Future Directions

This study presents data on concomitant IST use with eculizumab in a strictly defined population of patients with refractory AChR+ gMG who were recruited for REGAIN. Data on the real-world use of eculizumab and concomitant ISTs in AChR+ gMG are limited (29); however, recruitment has recently been initiated for the Registry of Participants with Generalized Myasthenia Gravis Treated with C5 Inhibition Therapies (www.clinicaltrials.gov: NCT04202341). This registry will collect data for up to 5 years from ~500 participants who are receiving, or who have received, Alexion C5 inhibition therapy, including details of concomitant IST use. These data will provide further information about IST use in patients with gMG treated with eculizumab and will extend the results of this study to reflect real-world clinical practice.

## Conclusions

IST use by patients with previously refractory gMG decreased in the REGAIN OLE. Importantly, clinical improvements were experienced by patients across IST change categories, including those who decreased and/or stopped IST. These results suggest that individuals with gMG who are treated with eculizumab may be able to successfully reduce their IST use, which is likely to ease their treatment-related burden. Individualized tapering of ISTs, guided by best practice standards, should therefore be considered in patients who respond to eculizumab.

## Data Availability Statement

The datasets presented in this article are not readily available. Alexion will consider requests for disclosure of clinical study participant-level data provided that participant privacy is assured through methods like data de-identification, pseudonymization, or anonymization (as required by applicable law), and if such disclosure was included in the relevant study informed consent form or similar documentation. Qualified academic investigators may request participant-level clinical data and supporting documents (statistical analysis plan and protocol) pertaining to Alexion-sponsored studies. Further details regarding data availability and instructions for requesting information are available in the Alexion Clinical Trials Disclosure and Transparency Policy at http://alexion.com/research-development. Requests to access the datasets should be directed to https://alexion.com/contact-alexion/medical-information.

## Ethics Statement

The studies involving human participants were reviewed and approved by ethics committees (see Appendix 2 in Supplementary Material). The patients/participants provided their written informed consent to participate in these studies.

## Author Contributions

RN, JH, MY, and FO'B contributed to the concept and design of the study. FO'B performed the statistical analyses. RN, SM, SB, FO'B, MY, and JH contributed to data acquisition, analysis or interpretation, drafting and critical revision, and final approval of the manuscript. All authors contributed to the article and approved the submitted version.

## Conflict of Interest

RN received research support from Alexion Pharmaceuticals, argenx, Genentech, Grifols, Immunovant, Momenta, the Myasthenia Gravis Foundation of America, the National Institutes of Health (National Institute of Neurological Disorders and Stroke and National Institute of Allergy and Infectious Diseases), and Ra Pharma and consultancy fees from Alexion Pharmaceuticals, argenx, CSL Behring, Grifols, Immunovant, Momenta, Ra Pharma, Roivant, and Viela Bio. SM received consultancy fees from Alexion Pharmaceuticals, argenx, and Ra Pharma. SB received research support from Alexion Pharmaceuticals, argenx, Catalyst, Mallinckrodt, Ra Pharma, and UCB Biosciences and consultancy and speaker fees from Akcea, Alexion Pharmaceuticals, Alnylam Pharmaceuticals, CSL Behring, Mitsubishi Tanabe, and Takeda. FO'B and MY are employed by and own stocks in Alexion Pharmaceuticals. JH received research support from Alexion Pharmaceuticals, argenx, the Muscular Dystrophy Association, and Ra Pharma; grants from the Centers for Disease Control and Prevention (Atlanta, GA, USA), the National Institutes of Health (including the National Institute of Neurological Disorders and Stroke and the National Institute of Arthritis and Musculoskeletal and Skin Diseases), and the Patient-Centered Outcomes Research Institute; consultancy fees from Alexion Pharmaceuticals, argenx, Ra Pharma, and Viela Bio; and non-financial support from argenx, Alexion Pharmaceuticals, and Ra Pharma.

## Abbreviations

AChR: acetylcholine receptor
AChR+: anti-acetylcholine receptor antibody-positive
AZA: azathioprine
gMG: generalized myasthenia gravis
IST: immunosuppressive therapy
MG: myasthenia gravis
MG-ADL: MG Activities of Daily Living
MMF: mycophenolate mofetil
OLE: open-label extension
PRED: prednisone and related corticosteroids
QMG: quantitative MG
SD: standard deviation.
