# Supplementary material for: Concomitant Immunosuppressive Therapy Use in Eculizumab-Treated Adults With Generalized Myasthenia Gravis During the REGAIN Open-Label Extension Study
Source: Front Neurol. 2020 Nov 24;11:556104. doi: 10.3389/fneur.2020.556104 (PMC7732596; doi:10.3389/fneur.2020.556104)
Supplement: Supplementary file 2 [file Data_Sheet_2.pdf]

Appendix 2: REGAIN and open-label extension study sites, ethics committee/institutional review board information

**Study site**	**Ethics committee/institutional review board (IRB)**
Instituto de Investigaciones Neurológicas Raúl Carrea (FLENI), Buenos Aires, Argentina	Comite de Etica en Investigaciones Biomedicas, Montañeses 2325, Ciudad Autonoma Buenos Aires, Buenos Aires C1428AQK, Argentina
Hospital Italiano de Buenos Aires, Buenos Aires, Argentina	Comité de Ética de Protocolos de Investigación, (CEPI), Juan. D Perón 4190/4192, C.A.B.A, Buenos Aires C1199AB/C1181ACH, Argentina
Algemeen Ziekenhuis Sint-Lucas – Campus Sint-Lucas, Ghent, Belgium	Ethisch Comité Universitair Ziekenhuis Antwerpen, Wilrijkstraat 10, 2650 Edegem, Belgium
	Commissie Medische Ethiek Algemeen Ziekenhuis Sint-Lucas, Groenebriel 1, 9000 Gent, Belgium
	Commissie Medische Ethiek van de Universitaire Ziekenhuizen K.U. Leuven, Campus Gasthuisberg E330, Herestraat 49, 3000 Leuven, Belgium
Universitair Ziekenhuis Antwerpen, Edegem, Antwerp, Belgium	Ethisch Comité Universitair Ziekenhuis Antwerpen, Wilrijkstraat 10, 2650 Edegem, Belgium
	Commissie Medische Ethiek Algemeen Ziekenhuis Sint-Lucas, Groenebriel 1, 9000 Gent, Belgium
	Commissie Medische Ethiek van de Universitaire Ziekenhuizen K.U. Leuven, Campus Gasthuisberg E330, Herestraat 49, 3000 Leuven, Belgium
Universitaire Ziekenhuizen Leuven, Leuven, Belgium	Ethisch Comité Universitair Ziekenhuis Antwerpen, Wilrijkstraat 10, 2650 Edegem, Belgium
	Commissie Medische Ethiek Algemeen Ziekenhuis Sint-Lucas, Groenebriel 1, 9000 Gent, Belgium
	Commissie Medische Ethiek van de Universitaire Ziekenhuizen K.U. Leuven, Campus Gasthuisberg E330, Herestraat 49, 3000 Leuven, Belgium
Faculdade de Medicina do ABC, Santo André, Brazil	Comité de Etica em Pesquisa da Faculdade de Medicinina do ABC, Avenida Lauro Gomes, 2000, Santo André, Sao Paulo 09060-870, Brasil
	Comissao Nacional de Etica em Pesquisa, Sepn 510 Norte, Bloco A 1º Subsolo, Edifício, Ex-Inan - Unidade II - Ministério da Saúde, Asa Norte Brasilia , DF 70750-521, Brasil
	SRTV 701, Via W 5 Norte, lote D, Edifício, PO 700, 3º andar, Asa Norte, CEP:70719-040, Brasilia/DF

Fundação Faculdade Regional de Medicina de São José do Rio Preto, São José do Rio Preto, Brazil	CEP da Faculdade de Medicina de São José do Rio Preto – FAMERP, Av. Brigadeiro Faria Lima, 5416, São José do Rio Preto, Sao Paulo 15090-000, Brasil
	Comissao Nacional de Etica em Pesquisa, Sepn 510 Norte, Bloco A 1º Subsolo, Edifício, Ex-Inan - Unidade II - Ministério da Saúde, Asa Norte Brasilia , DF 70750-521, Brasil
	SRTV 701, Via W 5 Norte, lote D, Edifício, PO 700, 3º andar, Asa Norte, CEP: 70719-040, Brasilia/DF
Hospital Mãe de Deus, Porto Alegre, Brazil	Comitê de Ética em Pesquisa em Seres, Humanos Associação Educadora São Carlos – Hospital Mae de Deus, Rua José de Alencar, 286 Porto Alegre, RS, 90880-480, Brasil
	Comissao Nacional de Etica em Pesquisa, Sepn 510 Norte, Bloco A 1º Subsolo, Edifício, Ex-Inan - Unidade II - Ministério da Saúde, Asa Norte, Brasilia, DF 70750-521, Brazil
	SRTV 701, Via W 5 Norte, lote D, Edifício, PO 700, 3º andar, Asa Norte, CEP: 70719-040, Brasilia/DF
Universidade Federal de São Paulo [UNIFESP], São Paulo, Brazil	CEP da Universidade Federal de São Paulo/Hospital São Paulo, Rua Botucatu, 572 - 1º andar, São Paulo, 04023-062, Brasil
	Comissao Nacional de Etica em Pesquisa, Sepn 510 Norte, Bloco A 1º Subsolo, Edifício, Ex-Inan - Unidade II - Ministério da Saúde, Asa Norte Brasilia , DF 70750-521, Brasil
	SRTV 701, Via W 5 Norte, lote D, Edifício, PO 700, 3º andar, Asa Norte, CEP: 70719-040, Brasilia/DF
University of Alberta Hospital, Edmonton, AB, Canada	University of Alberta Research Ethics Office, 308 Campus Tower, 8625 – 112 St. NW, Edmonton, Alberta T6G 1K8, Canada
Fakultní nemocnice Brno, Brno, Czech Republic	Eticka komise Fakultini nemocnice Brno, Jihlavska 20, 625 00 Brno, Czech Republic
Fakultní nemocnice Ostrava, Ostrava, Czech Republic	Eticka komise Fakultini nemocnice Ostrava, 17. listopadu 1790/5, 708 52 Ostrava-Poruba, Czech Republic
	Eticka komise Fakultini nemocnice Brno, Jihlavska 20, 625 00 Brno, Czech Republic
Všeobecná fakultní nemocnice v Praze, Praze, Czech Republic	Eticka komise Vseobecne fakultni nemocnice v Praze, Na Bojisti 1, III. patro 128 08, Praha 2, Czech Republic
	Eticka komise Fakultini nemocnice Brno, Jihlavska 20, 625 00 Brno, Czech Republic
Århus Universitetshospital, Aarhus, Denmark	De Videnskabsetiske Komitéer for Region, Hovedstaden, Kongens Vænge 2, Hillerød, 3400, Denmark
Rigshospitalet, Copenhagen, Denmark	De Videnskabsetiske Komitéer for Region, Hovedstaden, Kongens Vænge 2, Hillerød, 3400, Denmark

Neuro NEO Oy, Turku, Finland	Tukija, Valvira, Lintulahdenkuja, P.O. Box 210, Helsinki, FI-00531, Finland
Jahn Ferenc Dél-Pesti Kórház, Budapest, Hungary	Medical Research Council Ethics Committee for Clinical Pharmacology, Széchenyi István tér 7-8, Budapest, H-1051, Hungary
University of Szeged, Albert Szent-Györgyi Health Center, Szeged, Hungary	Medical Research Council Ethics Committee for Clinical Pharmacology, Széchenyi István tér 7-8, Budapest, H-1051, Hungary
Azienda Ospedaliera Sant'Andrea – Università di Roma La Sapienza, Rome, Italy	Comitato Etico Azienda Policlinico Umberto I, Viale del Policlinico 155, Roma, 00161, Italy
Azienda Ospedaliera Universitaria “Federico II”, Naples, Italy	Comitato Etico per le attività biomediche “Carlo Romano” Università degli Studi di Napoli Federico II, Via Pansini, 5, Napoli, 80131, Italy
Fondazione IRCCS Istituto Neurologico Carlo Besta, Milano, Italy	Comitato Etico Fondazione IRCCS Istituto, Neurologico “Carlo Besta”, Via Celoria 11, Milano, 20133, Italy
Policlinico Universitario Agostino Gemelli, Rome, Italy	Comitato Etico Univ. Cattolica del Sacro Cuore, Policlinico A. Gemelli, Largo Agostino Gemelli, 8, Roma, 00168, Italy
Umberto I Policlinico di Roma – Università di Roma La Sapienza, Rome, Italy	Comitato Etico dell' Universita Sapienza, Viale del Policlinico, 155, Roma, 00161, Italy
Chiba University Hospital, Chiba, Japan	Chiba University Hospital IRB, Chuo-ku Inohana 1-8-1, Chiba-shi, Chiba-Ken 260-8677, Japan
Kyushu University Hospital, Fukuoka	Kyushu University Hospital IRB, Higashi-ku Maidashi 3-1-1, Fukuoka-shi, Fukuoka-Ken 812-8582, Japan
Hanamaki General Hospital, Hanamaki, Japan	General Hanamaki Hospital IRB, Kajo-machi 4-28, Hanamaki-shi, Iwate-Ken 025-0075, Japan
Kinki University Hospital, Osaka, Japan	Kindai University Hospital IRB, Onohigashi 377-2, Osakasayama-shi, Osaka-Fu 589-8511, Japan
Nagasaki University Hospital, Nagasaki, Japan	Nagasaki University Hospital IRB, Sakamoto 1-7-1, Nagasaki-shi, Nagasaki-Ken 852-8501, Japan
National Hospital Organization Sendai Medical Center, Sendai, Japan	NHO Sendai Medical Center IRB, Miyagino-ku Miyagino 2-8-8, Sendai-shi, Miyagi-Ken 983-8520, Japan
Osaka University Hospital, Osaka, Japan	Osaka University Hospital IRB, Yamadaoka 2-15, Suita-shi, Osaka-Fu 565-0871, Japan
Sapporo Medical University Hospital, Chuo-ku, Sapporo, Japan	Sapporo Medical University Hospital IRB, 291, Minami 1jo Nishi 16-chome, Chuo-ku, Sapporo-shi, Hokkaido 060-8543, Japan
Korea University Anam Hospital, Seoul, Republic of Korea	IRB of Korea University Anam Hospital, 73, Inchon-ro, Seongbuk-gu, Seoul, 136-705, Korea

Samsung Medical Center, Seoul, Republic of Korea	IRB of Samsung Medical Center, 81, Irwon-ro, Gangnam-gu, Seoul, 135-710, Korea
Seoul National University Seoul Metropolitan Government Boramae Medical Center, Seoul, Republic of Korea	IRB of Seoul Metropolitan Government, Seoul National University Boramae Medical Center, 20, Boramae-ro 5-gil, Dongjak-gu, Seoul, 07061, Korea
Severance Hospital, Yonsei University Health System, Seoul, Republic of Korea	IRB of Severance Hospital, Yonsei University, Health System, 50-1, Yonsei-ro, Seodaemun-gu, Seoul, 03772, Korea
Academisch Medisch Centrum, Amsterdam, The Netherlands	Medisch Ethische Toetsingscommissie (MEC), Academisch Medisch Centrum Amsterdam, Kamernummer: E2-170/172, Meibergdreef 9, 1105 AZ Amsterdam, The Netherlands
Hospital Universitari de Bellvitge, Barcelona, Spain	CEIC Hospital Universitari Vall d’Hebron, Edificio metrno infntil planta 13, Paseo Vall d’Hebron 119-129, Barcelona 08035, Spain
	Comite Etico de Investigacion Clinica (CEIC), Hospital Universitàrio de Bellvitge, Edifici Unitat de Recerca Feixa Llarga, s/n L’Hospitalet de Llobregat, Barcelona, 08907, Spain
Hospital Universitario La Paz, Madrid, Spain	CEIC Hospital Universitàrio La Paz, Paseo de la Castellana, 261, Hospital General – Planta 8ª, 28046, Madrid, Spain
	CEIC Hospital Universitàrio de Bellvitge, Edifici Unitat de Recerca, Feixa Llarga, s/n, L’Hospitalet de Llobregat, Barcelona 08907, Spain
Hospital Universitari Vall d'Hebron, Barcelona, Spain	CEIC Hospital Universitari Vall d’Hebron, Edificio metrno infntil planta 13, Paseo Vall d’Hebron 119-129, Barcelona 08035, Spain
	CEIC Hospital Universitàrio de Bellvitge, Edifici Unitat de Recerca, Feixa Llarga, s/n, L’Hospitalet de Llobregat, Barcelona 08907, Spain
Hospital Sant Pau, Universitat Autònoma de Barcelona, Spain	Hospital de la Santa Creu I Sant Pau, Servicio de Farmacología Clínica – Pabellón, 18Av. Sant Antoni Mª Claret, 167, Barcelona, 08025, Spain
	CEIC Hospital Universitàrio de Bellvitge, Edifici Unitat de Recerca, Feixa Llarga, s/n, L’Hospitalet de Llobregat, Barcelona 08907, Spain
Karolinska University Hospital, Stockholm, Sweden	Regionala etikprövningsnämnden i Stockholm, Karolinska institutet/Solna, Nobels vägn 9, plan 3D, SE-171 65, Stockholm, Sweden
	Etikprövningsmyndigheten, Box 2110, 750 02 Uppsala, Sweden
Dokuz Eylül University Faculty of Medicine, Izmir, Turkey	Ege Üniversitesi Tip Fakültesi Klinik, Araştirmalar Etik Kurulu, Ege Üniversitesi Tip Fakültesi Dekanliği 2, Kat Erzene Ankara Caddesi, Bornova 35100, Izmir, Turkey
	Hacettepe Universitesi, Klinik Araştırmalar Etik, Kurulu Sıhhıye-Altındağ, Ankara/Turkey 06100

Hacettepe University Faculty of Medicine, Ankara, Turkey	Ege Üniversitesi Tip Fakültesi Klinik, Araştirmalar Etik Kurulu, Ege Üniversitesi Tip Fakültesi Dekanliği 2, Kat Erzene Ankara Caddesi, Bornova 35100, Izmir, Turkey
	Hacettepe Universitesi, Klinik Araştırmalar Etik, Kurulu Sıhhıye-Altındağ, Ankara/Turkey 06100
Kocaeli University Faculty of Medicine, Kocaeli, Turkey	Ege Üniversitesi Tip Fakültesi Klinik, Araştirmalar Etik Kurulu, Ege Üniversitesi Tip Fakültesi Dekanliği 2, Kat Erzene Ankara Caddesi, Bornova 35100, Izmir, Turkey
	Hacettepe Universitesi, Klinik Araştırmalar Etik, Kurulu Sıhhıye-Altındağ, Ankara/Turkey 06100
Ondokuz Mayis University Medical Faculty, Atakum/Samsun, Turkey	Ege Üniversitesi Tip Fakültesi Klinik, Araştirmalar Etik Kurulu, Ege Üniversitesi Tip Fakültesi Dekanliği 2, Kat Erzene Ankara Caddesi, Bornova 35100, Izmir, Turkey
	Hacettepe Universitesi, Klinik Araştırmalar Etik, Kurulu Tıp Fakültesi Dekanlık Katı Sıhhıye-Altındağ, Ankara/Turkey 06100
King’s College Hospital, London, UK	NRES Committee East Midlands – Leicester, The Old Chapel - Royal Standard Place, Nottingham, NG1 6FS, UK
Queen Elizabeth Neuroscience Centre, (Wellcome Trust CRF, University Hospitals Birmingham), Birmingham, UK	NRES Committee East Midlands – Leicester, The Old Chapel, Royal Standard Place, Nottingham, NG1 6FS, UK
The Walton Centre, Liverpool, UK	The Walton Centre NHS Foundation Trust, Clinical Trials Unit, 1st Floor, Lower Lane, Fazakerley Liverpool, Merseyside L9 7LJ, UK
	NRES Committee East Midlands – Leicester, The Old Chapel, Royal Standard Place, Nottingham, NG1 6FS, UK
Brigham and Women's Hospital, Boston, MA, USA	Partners Human Research Committee, 399 Revolution Dr, Suite 710, Somerville, MA 02145, USA
University at Buffalo Jacobs School of Medicine and Biomedical Sciences, Buffalo, NY, USA	Health Sciences IRB, 3435 Main Street - 150 Parker Hall, Buffalo, NY 14214, USA
California Pacific Medical Center, San Francisco, CA, USA	Western IRB (WIRB), 1019 39th Avenue SE Suite 120, Puyallup, WA 98374-2115, USA
Carolinas HealthCare System, Charlotte, NC, USA	Copernicus Group IRB, 1 Triangle Drive, Suite 100, Durham, NC 27713, USA
	Advarra IRB (Chesapeake IRB), 6940 Columbia Gateway Drive, Suite 110, Columbia, MD 21046, USA

Duke University Health System, Durham, NC, USA	Duke University Health System IRB, 2424 Erwin Road, Suite 405, Campus Box 2712, Durham, NC 27705, USA
Indiana University, Indianapolis, IN, USA	Indiana University IRB, 980 Lockfield Village, Third Floor, Indianapolis, IN 46202, USA
Johns Hopkins University School of Medicine, Baltimore, MD, USA	JHM Office of Human Subjects Research – IRBs, 1620 McElderry Street, Baltimore, MD 21205-1911, USA
Lahey Hospital and Medical Center – Burlington, Burlington, MA, USA	Lahey Hospital and Medical Center IRB, 41 Mall Road, Burlington, MA 01805, USA
Las Vegas Clinic, Las Vegas, NV, USA	Copernicus Group IRB, 500 CentreGreen Way, Suite 200, Cary, NC 27513, USA
Ohio State University Wexner Medical Center, Columbus, OH, USA	Western IRB (WIRB), 1019 39th Avenue SE Suite 120, Puyallup, WA 98374-2115, USA
Oregon Health and Science University, Portland, OR, USA	Oregon Health and Science University IRB, 3181 SW Sam Jackson Park Rd, Portland, OR 97239, USA
Southern Illinois University School of Medicine, Springfield, IL, USA	Springfield Committee For Research Involving Human Subjects, 201 East Madison Street, Springfield, IL 62702, USA
Stanford University School of Medicine, Stanford, CA, USA	Stanford IRB Research Compliance Office, 3000 El Camino Real, Five Palo Square, 4th Floor, Palo Alto, CA 94306, USA
The University of Texas Health Science Center at San Antonio, San Antonio, TX, USA	The University of Texas Health Science Center, San Antonio IRB, 7703 Floyd Curl Drive, San Antonio, TX 78229, USA
The University of Texas Southwestern Medical Center, Dallas, TX, USA	University of Texas Southwestern Medical Center IRB, 5323 Harry Hines Blvd., Dallas, TX 75390, USA
University of Alabama at Birmingham Medicine, Birmingham, AL, USA	Western IRB (WIRB), 1019 39th Avenue SE Suite 120, Puyallup, WA 98374-2115, USA
University of California-Irvine, Irvine, CA, USA	IRB – Office of Research Administration, University of California, Irvine, 141 Innovation, Suite #250, Irvine, CA 92697-7600, USA
University of California-San Francisco-Fresno, CA, USA	Community Medical Centers IRB, 155 N. Fresno Street, Suite 290, Fresno, CA 93701, USA
University of Florida Health Jacksonville, Jacksonville, FL, USA	Western IRB (WIRB), 1019 39th Avenue SE Suite 120, Puyallup, WA 98374-2115, USA
University of Iowa Children's Hospital, Iowa City, IA, USA	Western IRB (WIRB), 1019 39th Avenue SE Suite 120, Puyallup, WA 98374-2115, USA

University of Kansas Medical Center, Kansas City, KS, USA	Human Subjects Committee University of Kansas Medical Center, 3901 Rainbow Boulevard, Kansas City, KS 66160, USA
University of Maryland Medical Center, Baltimore, MD, USA	University of Maryland, Baltimore IRB, 800 W. Baltimore Street, Suite 100, Baltimore, MD 21201, USA
University of Miami Miller School of Medicine, Miami, FL, USA	University of Miami IRBs Human Subject Research Office, 1400 NW 10th Avenue, Suite 1200A, Miami, FL 33136, USA
University of North Carolina Medical Center, Chapel Hill, NC, USA	Office of Human Research Ethics, University of North Carolina at Chapel Hill, Medical School Building 52, CB# 7097, Chapel Hill, NC 27599, USA
University of South Florida, Tampa, FL, USA	Western IRB (WIRB), 1019 39th Avenue SE Suite 120, Puyallup, WA 98374-2115, USA
University of Southern California, Los Angeles, CA, USA	USC Health Sciences IRB, 1200 North State Street, Suite 4700, Los Angeles, CA 90033, USA
University of Vermont Medical Center, Burlington, VT, USA	University of Vermont Committee on Human Research in the Medical Sciences, 213 Waterman Building, 85 South Prospect Street, Burlington, VT 05405, USA
University of Washington, Seattle, WA, USA	Western IRB (WIRB), 1019 39th Avenue SE Suite 120, Puyallup, WA 98374-2115, USA
Wesley Neurology Clinic, PC, Cordova, TN, USA	Copernicus Group IRB, 1 Triangle Drive, Suite 100, Durham, NC 27713, USA
Yale University, New Haven, CT, USA	Human Research Protection Program, 25 Science Park, 3rd Floor, 150 Munson St., New Haven, CT 06520-8327, USA
